# Pulmonary Sclerosing Pneumocytoma: A Pre and Intraoperative Diagnostic Challenge. Report of Two Cases and Review of the Literature

**DOI:** 10.3390/medicina57060524

**Published:** 2021-05-23

**Authors:** Senia Maria Rosaria Trabucco, Debora Brascia, Gerardo Cazzato, Giulia De Iaco, Anna Colagrande, Francesca Signore, Giuseppe Ingravallo, Leonardo Resta, Giuseppe Marulli

**Affiliations:** 1 Pathology Unit, Department of Organ Transplantation and Emergency (DETO), University Hospital of Bari, 70124 Bari, Italy; xeniatrabucco@gmail.com (S.M.R.T.); anna.colagrande@gmail.com (A.C.); giuseppe.ingravallo@uniba.it (G.I.); leonardo.resta@uniba.it (L.R.); 2 Thoracic Surgery Unit, Department of Organ Transplantation and Emergency, University Hospital of Bari, 70124 Bari, Italy; deborabrascia@gmail.com (D.B.); g.deiaco24@gmail.com (G.D.I.); f.signore@studenti.uniba.it (F.S.)

**Keywords:** pulmonary sclerosing pneumocytoma, fine-needle aspiration, aspiration cytology, lung tumor

## Abstract

Pulmonary sclerosing pneumocytoma is a rare benign pulmonary tumor of primitive epithelial origin. Because of the unspecific radiological features mimicking malignancies and its histological heterogeneity, the differential diagnosis with adenocarcinoma and carcinoid tumors is still challenging. We report our experience of two cases of sclerosing pneumocytoma, as well as a review of the literature. Immunohistochemical findings showed intense staining of the cuboidal epithelial cells for cytokeratin-pool and TTF-1, with focal positivity for progesterone receptors. Round and spindle cells expressed positivity for vimentin, TTF-1 and focally for the progesterone receptor. Cytologic diagnosis of pulmonary pneumocytoma requires the identification of its dual cell population, made up of abundant stromal cells and fewer surface cells. Since the pre- and intraoperative diagnosis should guide surgical decision making, obtaining a sufficient specimen size to find representative material in the cell block is of paramount importance.

## 1. Introduction

Pulmonary sclerosing pneumocytoma (PSP), formerly known as pulmonary sclerosing hemangioma, is a rare pulmonary tumor initially described by Liebow et al. [1] in 1956, as a tumor with marked sclerosis and vascularization. PSP is usually seen in adults over 50 years old, featuring a female to male ratio of 5:1. PSP has been shown by immunohistochemical markers to have a primitive epithelial origin, most likely from type II alveolar pneumocytes. The essential feature of PSP is the presence of cuboidal surface cells and round stromal cells, both of which are thought to be neoplastic. In the 2015, the World Health Organization (WHO) classification “miscellaneous tumors” was changed to “adenomas” [2]. This rare benign disease may present some diagnostic challenges due both to radiological features mimicking malignancy, and to the fact that pre and postoperative cyto-histological investigations may depict characteristics similar to aggressive lung tumors. Here we present two interesting PSP cases and discuss the diagnostic evaluation and patient management, as well as a review of the more recent literature. Written informed consent to the publication of patient information and images was provided by the patients.

## 2. Materials and Methods

**Figure 1:**
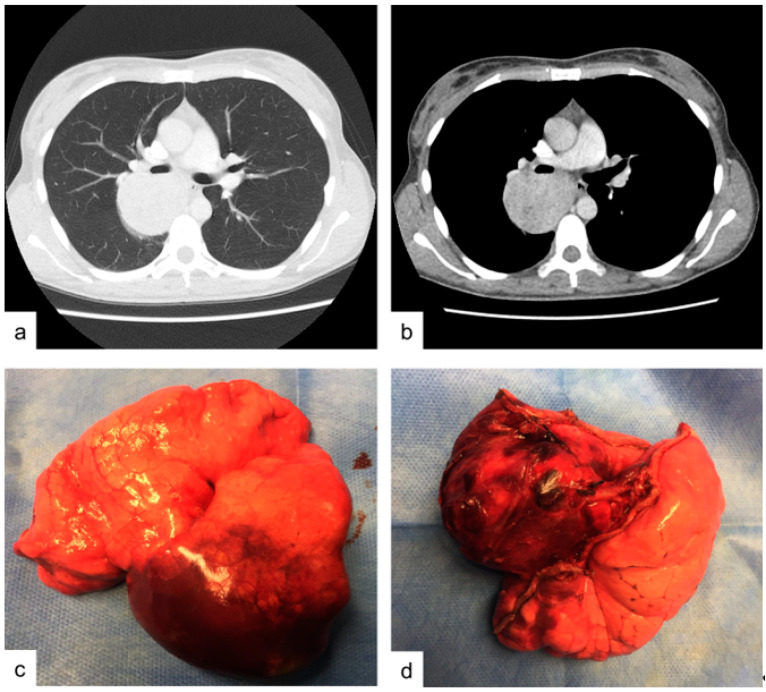
Case 1. (**a**,**b**) contrast enhanced chest CT-scan showing an hypodense soft-tissue lesion in the size of mm 50 by 65 mm located between the posterior segment of the right upper lobe and lower lobe upper segment. (**c**,**d**) operative specimen: right upper lobe and lower lobe upper segment after surgical excision.

**Figure 2:**
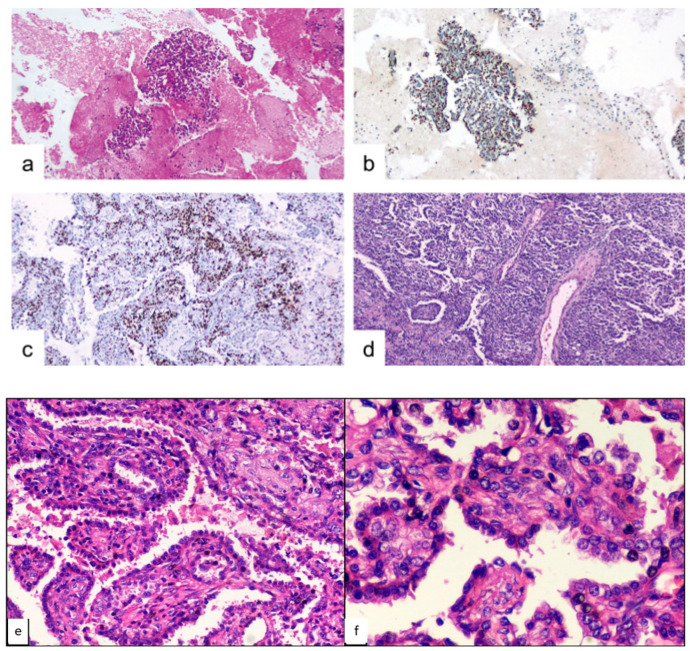
Case 1, microscopic findings. (**a**) cyto-inclusion: spindle cells with focal atypia in the adenomorphic and papillary pattern (hematoxylin-eosin, 10×). (**b**) cyto-inclusion: positivity of the neoplastic cells for TTF-1 (10×). (**c**) round and spindle cells aggregated in papillae solid nests ex-pressed focal positivity for progesterone-receptor (10×). (**d**) histological features of PSP, with varied proportions of sclerotic, solid, papillary and hemorrhagic patterns (hematoxylin-eosin, 10×). (**e**) histological detail of the PSP architecture (hematoxylin-eosin, 20×). (**f**) histological detail of the PSP architecture (hematoxylin-eosin, 40×).

A 38-year-old woman presented to the Emergency Department of our Hospital with rectorrhagia of uncertain origin and anemia. The patient denied hemoptysis, dyspnea, fever and weight loss. She had never smoked. Colonoscopy showed diffuse polyposis throughout the bowel. While performing routine pre-procedural analysis before colonoscopy, chest X-rays showed a huge abnormal mass occupying the right upper pulmonary lobe. Contrast-enhanced chest CT scan showed a well-defined, hypodense soft-tissue lesion measuring 50 by 65 mm, located in the posterior segment of the right upper lobe and upper segment of the right lower lobe, in contiguity with the adjacent mediastinal pleura (Figure 1a,b). Positron emission tomography PET/CT scan revealed slight fluorine-18 deoxyglucose (FDG) uptake within the lesion (SUV max: 3.5). Endobronchial ultrasound-guided transbronchial needle aspiration (EBUS-TBNA) was performed using a 22 G aspiration needle (Vizishot^®^, Olympus, Japan). The cytologic preparation showed epithelial clusters of monomorphic cells with focal atypia in an adenomorphic and papillary pattern, without mitosis. These findings were highly suggestive of a neuroendocrine pulmonary tumor. However, immunohistochemistry revealed positive staining for CK-7 and TTF-1, while common neuroendocrine markers such as chromogranin A and synaptophysin were negative, excluding the diagnosis of a carcinoid tumor. The Proliferative index, evaluated by Ki67+, was <2–3%. The final cytological report supported a diagnosis of lung adenocarcinoma (shown in Figure 2a,b). For this reason, the patient underwent surgery: through a lateral right thoracotomy, right upper lobectomy plus lower lobe upper segmentectomy (due to the suspicion of transfissural invasion) were performed.

### 2.1. Macroscopic Findings.

The resected specimen contained a yellowish tumor, easily enucleable, with central necrosis, measuring 5.5 by 5.0 cm in diameter (Figure 1c,d). The tumor markedly expanded the lungs, was encapsulated, peripheral and subpleural.

### 2.2. Microscopic Findings

Neoplasm with a predominantly solid (80%) and partly papillary/sclerotic (20%) pattern. The solid areas consist of round and spindle-shaped stromal cells organized in clusters and some more clearly epithelial elements, also aggregated in nests of various sizes. The papillary component often includes sclerotic fibro-connective axes comprising round and spindle cells, covered with cuboidal, monofilament epithelial elements. A few rare mitoses and only focal cellular atypia’s are present. There is no sign of vascular invasion (Figure 2b,c).

## 3. Case Report 2

**Figure 3:**
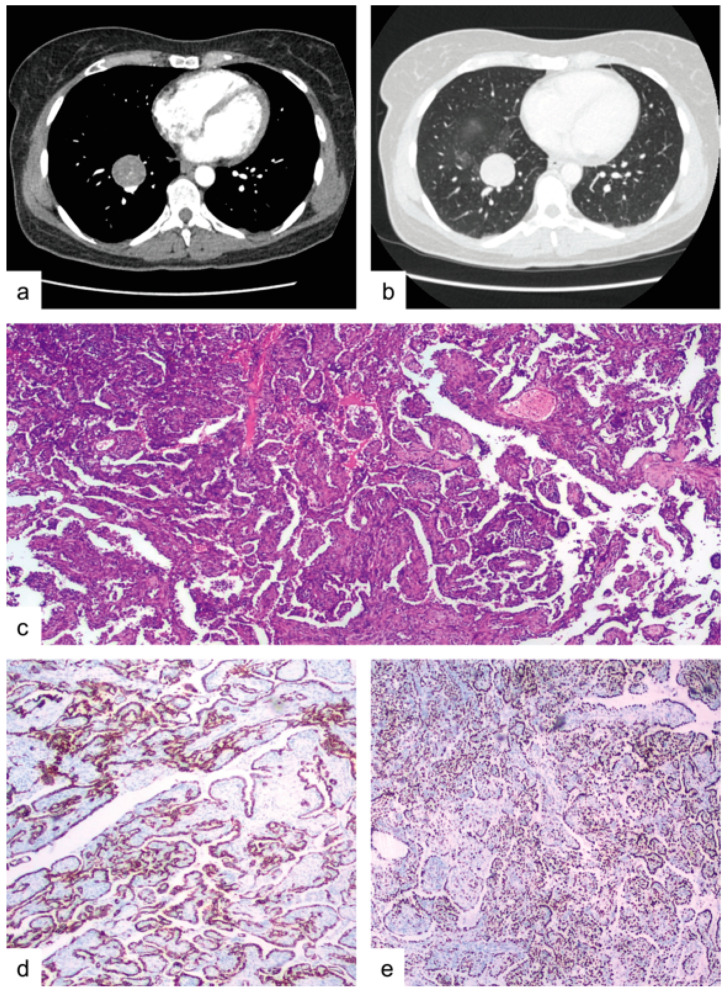
Case 2. (**a**) contrast enhanced chest CT-scan revealing a 35 mm in diameter oval-shaped solid mass with well-defined borders in the right lower lobe, medial basal segment. (**b**) the above-mentioned mass with surrounding ground-glass opacities. (**c**) histological features of PSP, including both cell types (epithelial and stromal) aggregated mainly in a papillary/sclerotic pattern (85%) with a smaller solid pattern component (15%), (hematoxylin-eosin, 10×). (**d**) positivity of the neo-plastic cells for CK-7 (10×). (**e**) positivity of the majority of the spindle cells for TTF-1 (10×).

A 48-year-old female patient presented to our University Hospital with a recent diagnosis of high-grade infiltrating breast carcinoma, scheduled to undergo a right lateral external quadrantectomy. She denied hemoptysis, dyspnea, fever and weight loss. She had never smoked. During pre-operative investigations, the patient underwent a total-body CT scan, which revealed an oval-shaped solid mass, 35 mm in diameter, with well-defined borders, in the medial basal segment of the right lower lobe, there were also surrounding ground-glass opacities, located in the right upper and lower lobes (Figure 3a,b). PET-CT scan showed slight uptake in the nodule (SUV max: 3.4). Radiological findings were not conclusive, and a malignancy could not be excluded, so the patient was a candidate for surgical resection of the mass, the extent to be decided on the basis of the intraoperative histological results. Frozen samples were sent to the Department of Pathology during the surgery and yielded positive results for an epithelial tumor of uncertain origin. Therefore, the patient underwent video-assisted thoracic surgery (VATS) consisting of right lower lobectomy and systematic lymphadenectomy. The resected specimen included a roundish, lobulated mass with a maximum diameter of 3.5 cm, whitish in color, with a tense-elastic consistency; it was encapsulated with respect to the surrounding parenchyma and occupied the middle basal segment of the lower right lobe. This lesion was 0.7 cm from the pleura and 4.5 cm from the bronchial surgical resection margin. The neoplasm consisted of both cell types (epithelial and stromal) aggregated mainly in a papillary/ sclerotic pattern (85%), with a smaller solid pattern component (15%) (Figure 3c). The cuboidal epithelial cells that line the papillae showed intense staining for cytokeratin-pool, cytokeratin 7 and TTF-1 but were negative for vimentin; there was focal positivity for the progesterone receptor. Round and spindle cells, aggregated in solid nests in the papillae, were positive for vimentin, thyroid transcription factor-1 (TTF-1) and focally for the progesterone receptor, and negative for cytokeratin-pool and cytokeratin 7 (Figure 3d,e). The Ki67 proliferative index was <5%, except for the solid pattern where it was increased by 10%.

Video-assisted thoracic surgery (VATS) right lower lobectomy and systematic lymphadenectomy. The resected specimen included a roundish, lobulated mass, with a maximum diameter of 3.5 cm, of whitish color, tense-elastic consistency, encapsulated with respect to the surrounding parenchyma, and that occupied the middle basal segment of the lower right lobe. This lesion was 0.7 cm from the pleura and 4.5 cm from the bronchial surgical resection margin. Neoplasm consisting of both cell types (epithelial and stromal) aggregated mainly in a papillary/sclerotic pattern (85%) with a smaller solid pattern component (15%) (shown in Figure 3c). The cubic epithelial cells that line the papillae, intensely stained with cytokeratin-pool, cytokeratin 7 and TTF-1, and were negative for vimentin; focal positivity for progesterone receptor. Round and spindle cells aggregated in papillae solid nests expressed positivity for vimentin, thyroid transcription factor-1 (TTF-1) and focal progesterone-receptor and were negative for cytokeratin-pool and cytokeratin 7 (shown in Figure 3d,e). The proliferative-index Ki67 was <5%, except for the solid pattern in which an increase of 10% was observed.

## 4. Discussion

PSPs are rare pulmonary neoplasms classified as adenomas in the latest WHO classification. [2] Over the last years, many studies have discussed the origin, differential diagnosis, potential malignancy and best treatment option for these rare tumors. For many years, in fact, this tumor was presumed to be of vascular origin. Instead, immunohistochemical results, in particular the TTF-1 expression, confirm its epithelial origin from the respiratory epithelium [3]. As in our cases, PSPs are often asymptomatic, incidental findings; rarely they present with parenchymal compression or thoracic pain, since they can grow to several centimeters [3]. Radiological findings can support the diagnosis. PSPs, in fact, present as a peripheral, solitary, well-defined mass, more frequently located in the lower lobes, with overlying vessels (26.3% of cases) [4] and surrounding ground-glass opacities [5]. FDG PET/CT scan has demonstrated a low to moderate uptake in PSPs. PSPs have a low malignant potential, good prognosis and in almost all cases, wedge resection or enucleation alone are sufficient for treatment. In 1% of cases, regional lymph node metastasis can be found, but this does not affect prognosis [4].

Pre-operative diagnosis, when feasible, is helpful to plan the best therapeutic strategy. It is well known that PSP is characterized by two different cell populations, surface and round stromal cells, typically organized in four architectural patterns: papillary, sclerotic, solid and hemorrhagic [5,6,7,8,9]. The cytological features parallel these major histological patterns; at least two (more frequently the papillary and solid patterns) of the four architectural patterns occur in all patients, and three of the patterns are found in 95% of patients [10]. Gal et al. [11] was the first to report that the cytologic diagnosis of PSP requires the identification of its dual cell population, made up of abundant stromal cells and fewer surface cells. The characteristics have been widely described in literature and are summarized in Table 1. The background, which is often hemorrhagic and without necrosis, could also guide diagnosis when numerous foamy macrophages, histiocytes and respiratory ciliated cells are observed. The problem is that fine-needle aspiration (FNA) cytology is poorly diagnostic, both due to the rarely distinct separation of the two tumor cell types in the specimen and to the disease rarity and hence potential unfamiliarity with its cytologic features of most pathologists. Preoperative cytologic and histological findings of PSP are limited to few case reports, in which computed tomography (CT)-guided FNA, EBUS-TBNA or intraoperative frozen sections (FS) often led to misdiagnosis. In any case, the histologic heterogeneity of this tumor makes the diagnosis challenging because, depending on the needle biopsy sampling area, the smears can vary from hypocellular, bloody, sclerotic to hypercellular, loaded with stromal fragments and/or showing epithelial cell proliferation [12]. In this context, finding representative material in the cell block is of paramount importance, allowing both immunohistochemistry and the recognition of different cell patterns [11,13,14]. In fact, while papillary groups are often seen in the cytologic preparations, the sclerotic and the hemorrhagic pattern are not frequently present in aspiration samples [13] As Shiba et al. [15] reported, cytological diagnosis could be easier for tumors with a predominantly papillary growth than with a solid pattern. Sclerosing hemangioma has a unique immunohistochemical profile which could guide differentiation between the two cell populations. Both cell types, in fact, usually show diffuse positivity for TTF-1, vimentin and epithelial membrane antigen (EMA) and negative staining for neuroendocrine markers. Moreover, the surface cells usually stain positive for napsin-A, cytokeratin AE1/AE3, CK7, CAM 5.2 and surfactant proteins A and B, while they are negative for ER, PR, CK20, CK5/6, S-100, SMA, calretinin and neuroendocrine markers. In contrast, the round stromal cells stain positive for PR and ER, focally for CAM 5.2 and CK7, while they are usually negative for AE1/AE3 and surfactant markers, pancytokeratin and Clara cell antigen. The Ki-67 labeling index expression is usually <5% in sclerosing pneumocytoma [12,16,17]. Interestingly, in our cases, the Ki67 proliferative index was <5% except in the solid pattern where it was increased by 10%. This led us to hypothesize that the papillary pattern could represent the benign component, and the sclerosing pattern could characterize the regressive aspects of the neoplasm. On the other hand, the solid growth pattern could represent the hormone-related component with the highest proliferative fraction, responsible for the neoplastic growth and the malignant potential, albeit low, of these neoplasms. The synchronous occurrence of breast cancer and PSP in our second case has rarely been described in literature, and in these rare cases is often considered coincidental. The problem linked to their synchronous presence is making a differential diagnosis between PSP and breast cancer metastasis, considering their similar radiological characteristics. In this context, PSP positive staining for ER and PR receptors may be interpreted as a possible metastasis, especially when the diagnosis is made by fine needle aspiration biopsy and TTF-1 staining is not performed due to limited biopsy material. It is important to be aware of this possibility and staining for TTF-1 should always be included in the immunohistochemical analysis in all cases with minimal or no atypia [18]. Preoperative cytologic and histological findings of PSP are limited to few case reports, in which computed tomography (CT)-guided FNA, EBUS-TBNA or intraoperative frozen sections (FS) often led to misdiagnosis. Table 2 and Table 3 summarize cytological findings described in literature since 2010 after CT-guided FNA [12,14,18,19,20,21,22,23,24] and EBUS-TBNA [25,26], respectively. In almost all cases of preoperative CT-guided FNA, both stromal and surface cells, organized in at least two architectural patterns, were found. The hemorrhagic background and absence of necrosis, or else the presence of mitosis, guided the diagnosis, along with the typical immunohistochemical pattern. As regards EBUS-TBNA cytological findings, only two case reports have been published to date, excluding the present case. While Shiina et al. [25] succeeded in recognizing both surface and stromal cells showing their typical immunohistochemical pattern for PSP and achieved diagnosis, Kosmas et al. [26] only found bronchial monomorphic cells with focal atypia, which led to misdiagnosis, as in our case. Moreover, the case described by Kosmas also showed negative staining for TTF-1 and napsin-A, which led the pathologists to suspect a malignant neoplasm of uncertain origin. These reports prove that EBUS-TBNA alone is poorly diagnostic for this tumor, especially if the specimen size is not sufficient to allow accurate histological examination and complete immunohistochemical analysis. In this setting, rapid on-site evaluation (ROSE) could have a role in increasing ultrasound-guided transbronchial needle aspiration accuracy.

Cytological heterogeneous findings also complicate the differential diagnosis with adenocarcinomas and carcinoid tumors. A papillary pattern, in fact, could also be found in well-differentiated lung adenocarcinomas (particularly the lepidic type) and in pulmonary carcinoid tumors. In these cases, the morphology of cells and nuclei has a leading role. Compared to sclerosing hemangioma, well-differentiated papillary adenocarcinoma often shows necrosis, abnormal nuclear shapes, a higher N/C ratio and notching. In contrast, the presence of a homogeneous population of cells with salt and pepper nuclear chromatin, and lacking hyalinized stroma, helps to differentiate it from PSP [17]. Diagnostic difficulties may arise when (1) there is cytological atypia, (2) one pattern predominates or (3) immunohistochemical analysis is non-conclusive due to insufficient material. As in our first case, in fact, TTF-1 staining alone often leads to misdiagnosis since it is not specific and may be present in primary adenocarcinomas, carcinoid tumors and metastatic thyroid carcinomas [11].

Differential diagnosis is important, especially during intraoperative analysis, and accurate analysis of frozen sections to guide the correct surgical treatment. Table 4 summarizes PSP histological frozen section findings described in literature since 2010 [27,28,29,30,31]. It is clear that frozen sections often lead to misdiagnosis, as occurred in our second case. The most helpful diagnostic clues in FS are the tumor circumscription and identification of various histological growth patterns [32]. In most cases, two or more of these patterns are present, but frequently one is predominant, leading to misdiagnosis; additional histological patterns not revealed at frozen section are often later identified in the paraffin sections.

## 5. Conclusions

In conclusion, a knowledge of this rare type of neoplasm is fundamental, for both surgeons and pathologists. Differential diagnosis can be challenging due to the lack of clinical and radiological support, and to unfamiliarity with the histological and immunohistopathological characteristics of these rare tumors. Since the pre- and intraoperative diagnosis should guide surgical decision making, obtaining a sufficient specimen size to find representative material in the cell block is of paramount importance.

## Figures and Tables

**Table 1:** Cytological findings of stromal and surface cells in pulmonary sclerosing pneumocytoma.

	Stromal Cuboidal/Round Cells	Surface Cells
Size	small to intermediate	large
Architecture	papillary clusters or flat sheets	pavement-type pattern, resembling pneumocytes
Cytoplasm	moderate/abundant eosinophilic cytoplasm	foamy cytoplasm
Nuclei	round to oval bland nuclei, nuclear pleomorphism	intranuclear inclusions, coarser chromatin
Nucleoli	inconspicuous nucleoli	small and occasional
N/C ratio	high	low

**Table 2:** Summary of the PSP cytological findings in CT-guided FNAs described in Literature in the last 10 years.

Author	Year	Cases	Final Cytologic Diagnosis	No. of Patterns	Hemorragic Background	Mytosis/Necrosis	IHC								
							Vimentin	CK7	EMA	TTF-1	PR	Napsin-A	ER	CK AE1/AE3	Ki67
Dettrick [14]	2012	1	**PSP**	3	Y	N	+ (both)	+ (surface)	+ (both)	+ (both)	+ (both)	+ (surface)		+	low
Saha [19]	2013	1	AC	3	Y	/				+ (both)					<5%
Salemis [18]	2013	1	**PSP**	3	/	/	+ (both)		+ (both)	+ (both)	+ (stromal)		+ (stromal)		
Onorati [20]	2016	1	**PSP**	3	Y	N			+ (both)	+ (both)				+ (surface)	5%
Zeng [12]	2016	1	**PSP**	3	Y	N				+ (both)	+ (stromal)			+ (surface)	<3%
Hissong [21]	2017	1	AC	3	Y	/		+ (surface)		+ (both)		+ (surface)			
Zhou [22]	2017	1	AC	2	/	/				+ (both)		+ (surface)			
Lee [23]	2019	1	**PSP**	4	/	/		+ (surface)		+ (both)		+ (surface)			
Sakai [24]	2019	1	**PSP**	2	/	/				+ (both)					<5%
Le [25]	2020	1	**PSP**	4	/	/	+ (both)			+ (both)	+ (stromal)		-	+ (surface)	<1%

PSP: Pulmonary Sclerosing Pneumocytoma; AC: Adenocarcinoma; IHC: Immunohistochemical.

**Table 3:** Summary of the PSP cytological findings in EBUS-TBNA described in Literature in the last 10 years.

Author	Year	Cases	ROSE	Final Cytologic Diagnosis	IHC							
					Vimentin	CK7	TTF-1	PR	Napsin-A	Neuroendocrine Markers	p63	Ki67
Shiina [26]	2018	1	N	**PSP**	+ (stromal)		+ (both)	+ (both)	+ (surface)			
Kosmas [27]	2020	1	N	SM			-		-		-	
Our case	2020	1	N	AC		+ (both)	+ (both)			*-*		*<3%*

PSP: Pulmonary Sclerosing Pneumocytoma; AC: Adenocarcinoma; SM: suspected malignancy; IHC: Immunohistochemical.

**Table 4:** Summary of the PSP cytological findings in frozen sections described in literature in the last 10 years.

Author	Year	Cases	Final Diagnosis	No. of Patterns	Mytosis/Necrosis	Growth Patterns				Hemorragic Background
						Papillary	Sclerosing	Solid	Hemorragic	
Blanco [28]	2011	2	**PSP**	2	N	+	-	-	+	Y
Kuroda [29]	2016	3	**PSP** (3)	2	/	+	-	+	-	/
Wu [30]	2016	14	**PSP** (7) AC (1) Benign (3) Deferred (3)	2	N	+	-	+	-	/
Yang [31]	2017	59	**PSP** (26) Malignancy (10) Benign (14) Deferred (9)	2 to 4	N	+ (10)	+ (14)	+ (21)	+ (14)	/
Cai [32]	2020	1	**PSP**	3	N	+	+	-	+	Y

PSP: Pulmonary Sclerosing Pneumocytoma; Ha: Hamartoma; AC: Adenocarcinoma; BAC: Bronchioloalveolar carcinoma; ASC: Adenosquamous carcinom.
